# Lung Compliance in a Case Series of Four COVID-19 Patients at a Rural Institution

**DOI:** 10.7759/cureus.9472

**Published:** 2020-07-30

**Authors:** David Lucero, Shobha Mandal, Apurwa Karki

**Affiliations:** 1 Internal Medicine, Guthrie Robert Packer Hospital, Sayre, USA; 2 Critical Care Medicine, Guthrie Robert Packer Hospital, Sayre, USA

**Keywords:** covid 19, covid-19, pulmonary critical care, ards, compliance

## Abstract

The severe acute respiratory syndrome coronavirus 2 (SARS-CoV-2) epidemic has generated a plethora of scientific articles. One interesting aspect of the virus is the binary phenotypic presentation in patients. While patients might meet the Berlin criteria for acute respiratory distress syndrome (ARDS), not all patients experience the same decrease in lung compliance as typically seen with ARDS. We have observed patients meeting ARDS criteria with higher lung compliance as measured through peak pressures at our institution. This phenotype difference is important with regard to how the patients are managed. Lower positive end-expiratory pressure (PEEP) and higher tidal volumes can be used in this phenotype.

## Introduction

Severe acute respiratory syndrome coronavirus 2 (SARS-CoV-2) is a virus that predominantly causes respiratory illness resulting in coronavirus disease 2019 (COVID-19)^ ^[1]. The disease was declared a global pandemic on March 11, 2020, by the World Health Organization (WHO) and has caused 202,000 deaths globally as of April 27, 2020, according to WHO statistics. SARS-CoV-2 belongs to the beta coronavirus genus, which includes severe acute respiratory syndrome coronavirus 1 (SARS-CoV-1), Middle East respiratory syndrome-related coronavirus (MERS-CoV) and two other human coronaviruses: human coronavirus OC43 (HCoV-OC43) and Human coronavirus HKU1 (HCoV-HKU1). The case fatality rate (CFR) of SARS-CoV-2 is 0.6%-3.5%, which is lower compared to that of SARS-CoV-1 and MERS-CoV where the CFR is 9 and 36% respectively [2,3]. The majority of the patients who have died of COVID-19 had acute respiratory distress syndrome (ARDS) [4]. Guideline-related recommendations for the management of ARDS in patients with COVID-19 propose invasive mechanical ventilation with low tidal volume (4-6 ml/kg of body weight) with a higher positive end-expiratory pressure (PEEP) strategy [5]. However, there is an evolving hypothesis related to different phenotypes in COVID-19-associated ARDS, and one of the phenotypes may be associated with high lung compliance [6]. There is a concern with the use of a high PEEP strategy for invasive mechanical ventilation in these patients with high lung compliance [7]. It has been suggested that normal compliance is more common [8]. One clinical implication of normal compliance is that higher tidal volumes [7-8 ml/kg of ideal body weight (IBW)] can be tolerated and it may improve dyspnea. Other institutions have classified similar phenotypes based on severity. Marini et al. have used type L and type H to describe high-compliant less severe disease, and lower compliant higher severity disease, respectively [9].

## Case presentation

**Figure 1:**
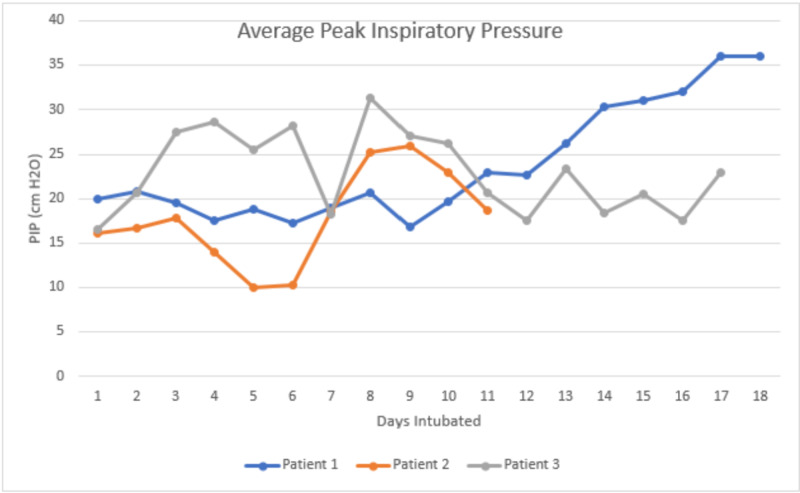
Average peak inspiratory pressure while intubated PIP: peak inspiratory pressure

**Figure 2:**
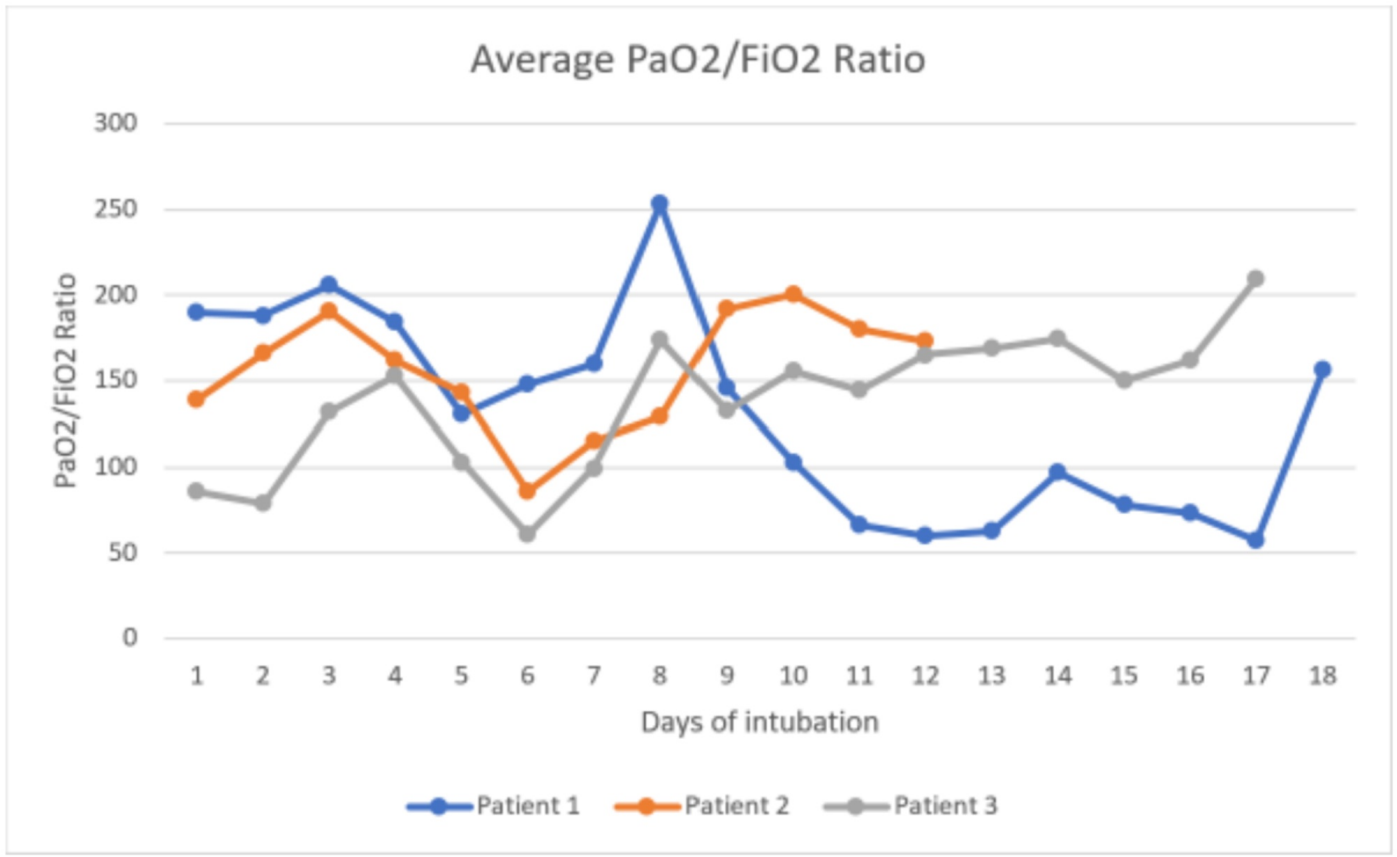
Average PaO2/FiO2 while intubated PaO2: partial pressure of oxygen; FiO2: fraction of inspired oxygen

We present a case series of four patients with confirmed COVIID-19 admitted to our hospital, with a focus on lung compliance. Three of the four patients required intubation, while the fourth passed away before intubation. Average peak pressure ranged from 10 to 36 cm H2O (Figure 1). The highest peak pressure was seen in patient 1 towards the end of life on airway pressure release ventilation (APRV). This would suggest that unlike traditional ARDS, our patients experienced a COVID-19 phenotype with higher compliance. As compliance decreases, the response to PEEP increases. It is important to note that the disease exists on a spectrum. All of our patients fit the Berlin criteria for ARDS. Figure 2 illustrates the average partial pressure of oxygen to fraction of inspired oxygen (PaO2:FiO2) ratio intubated each day.

In our case series, as patient 1 continued to deteriorate, the ventilation strategy evolved. The patient was started on non-invasive ventilation (NIV) and was transitioned to pressure-regulated volume control (PRVC) with increasing FiO2 and PEEP requirements. On day 10 of admission, the patient was started on APRV. Nitrous oxide was started on day 11 of admission. On day 16, the patient was transitioned back to PRVC but required APRV after one day. O2 saturation ranged from 82% to 97.1%. The highest average peak inspiratory pressure reached 36 cm H2O while the patient was on APRV [high pressure (PHigh): 32, high time (THigh): 4.5, PEEP: 0, TPEEP: 0.5, and 100% inhaled nitrous oxide (iNO): 60 ppm]. PaO2:FiO2 ratio ranged from 253 to 55. Patient 2 was started on continuous positive airway pressure (CPAP) and transitioned to PRVC. On day eight of hospitalization, the patient was transitioned to pressure support ventilation (PSV). By day 10, the patient was tolerating nasal cannula for one day before being transitioned back to NIV. On day 14, they once again required PRVC. The maximum average peak inspiratory pressure reached 26 cm H2O on PRVC [tidal volume (Vt): 550 ml, rate: 20, FiO2: 60%, and expiratory positive airway pressure (EPAP): 14 cm H20]. PaO2:FiO2 ratio ranged from 200 to 70. Patient 3 was transferred to our hospital intubated. The patient was on PRVC when admitted. He self-extubated and was trialed on non-rebreather (NRB); however, he required re-intubation. PEEP was increased; however, O2 sat remained in the 80s, and on day two, he was started on APRV. On day three, he was transitioned back to PRVC. On day six, he was re-started on APRV with iNO. He was switched to PRVC with iNO on the same day. He continued on PRVC until his family decided to make the patient's status comfort care on day 17, and he subsequently passed away. The maximum average peak inspiratory pressure reached 27.5 cm H2O on APRV (PHigh: 20, THigh: 4.0, PEEP: 0, TPEEP: 0.5, and 70% FiO2). PaO2:FiO2 ratio ranged from 229 to 57. Patient 4 declined to be intubated. The patient was Do-Not-Intubate (DNI). She was escalated from nasal cannula to high-flow non-rebreather. It is worth noting that the patient was not initially endorsing dyspnea on nasal cannula. The day after the patient changed her code status, she decompensated and passed away.

## Discussion

COVID-19 disease is a new entity that causes ARDS and has a remarkable array of presentations with symptoms related to the respiratory system. The presentation may vary between normal breathing to profound dyspnea, hypocarbia or hypercarbia, response or lack thereof to pulmonary vasodilators and prone positioning [7]. However, in one study, over 50% of patients were found to have near-normal lung compliance [7]. The possible etiology of preserved lung compliance could be secondary to pulmonary vasoplegia and the resulting shunting of the blood flow away from the well-ventilated regions of the lung [6]. The use of a high PEEP strategy may result in hemodynamic adverse effects without any accompanying benefit in the management of hypoxemia [7]. There is a theoretical benefit of trying to avoid any effect of barotrauma by invasively ventilating patients with COVID-19-related respiratory failure.

## Conclusions

Our case series fits with the current observation that while COVID-19 may present with an ARDS-type response, the lungs can in fact retain compliance. In our case series, the patients presented as “Type 1” or “Type L” as evidenced by the relatively low peak inspiratory pressures.
